# Rapidly acquired multisensory association in the olfactory cortex

**DOI:** 10.1002/brb3.390

**Published:** 2015-10-14

**Authors:** Prasanna R. Karunanayaka, Donald A. Wilson, Megha Vasavada, Jianli Wang, Brittany Martinez, Michael J. Tobia, Lan Kong, Paul Eslinger, Qing X. Yang

**Affiliations:** ^1^ Department of Radiology (Center for NMR Research)The Pennsylvania State University College of MedicineHersheyPennsylvania; ^2^ Emotional Brain InstituteNathan S. Kline Institute for Psychiatric ResearchOrangeburgNew York; ^3^ Department of Child & Adolescent PsychiatryNew York University School of MedicineNew York CityNew York; ^4^ Department of Public Health SciencesThe Pennsylvania State University College of MedicineHersheyPennsylvania; ^5^ Department of NeurologyThe Pennsylvania State University College of MedicineHersheyPennsylvania; ^6^ Department of NeurosurgeryThe Pennsylvania State University College of MedicineHersheyPennsylvania

**Keywords:** associative learning, fMRI, ICA, odor intensity, unified SEM

## Abstract

**Background:**

The formation of an odor percept in humans is strongly associated with visual information. However, much less is known about the roles of learning and memory in shaping the multisensory nature of odor representations in the brain.

**Method:**

The dynamics of odor and visual association in olfaction was investigated using three functional magnetic resonance imaging (fMRI) paradigms. In two paradigms, a visual cue was paired with an odor. In the third, the same visual cue was never paired with an odor. In this experimental design, if the visual cue was not influenced by odor–visual pairing, then the blood‐oxygen‐level‐dependent (BOLD) signal elicited by subsequent visual cues should be similar across all three paradigms. Additionally, intensity, a major dimension of odor perception, was used as a modulator of associative learning which was characterized in terms of the spatiotemporal behavior of the BOLD signal in olfactory structures.

**Results:**

A single odor–visual pairing cue could subsequently induce primary olfactory cortex activity when only the visual cue was presented. This activity was intensity dependent and was also detected in secondary olfactory structures and hippocampus.

**Conclusion:**

This study provides evidence for a rapid learning response in the olfactory system by a visual cue following odor and visual cue pairing. The novel data and paradigms suggest new avenues to explore the dynamics of odor learning and multisensory representations that contribute to the construction of a unified odor percept in the human brain.

## Introduction

Odor perception is highly influenced by knowledge, experience, internal states, and sensory information from other modalities (Wilson and Stevenson 2006). Specifically, in humans, odor discriminability and odor identification can be improved by familiarity (Rabin and Cain 1984; Stevenson 2001; Li et al. 2006), and odor detection and perceived intensity can be modulated by congruent visual cues (Gottfried and Dolan 2003; Osterbauer et al. 2005; Stevenson and Oaten 2008; Zhou et al. 2012). For example, the detection threshold is reduced and perceptual intensity enhanced of a cherry odor if it is delivered in a red liquid compared to the same odor delivered in a clear liquid (Zellner et al. 1991; Shankar et al. 2010).

The link between odors and multisensory cues involves associative memory, and may be subserved by a semantic and a relatively pure sensory component (Royet et al. 1999; Koenig et al. 2000; Olsson and Friden 2001; Olsson et al. 2002; Gottfried et al. 2004; Yeshurun et al. 2008; Zelano et al. 2009; Smeets and Dijksterhuis 2014). Olfactory sensory memory reflects activity, in part, within the primary olfactory cortex (POC), which like most sensory cortices (Ghazanfar and Schroeder 2006) receives direct and/or indirect convergent multisensory inputs (Gottfried et al. 2004; Wesson and Wilson 2010; Maier et al. 2012). For example, fMRI studies of odor–visual association (or integration) have shown that a visual cue previously associated with an odor can, in the absence of odor stimulation, elicit neural activity in the primary olfactory cortex (Gottfried et al. 2003, 2004). In this paradigm, stimulus‐evoked activity within the hippocampus and orbitofrontal cortex was also shown to be experience dependent (Gottfried et al. 2003). Importantly, olfactory associative memory can occur very quickly, in some cases with a single trial (Sullivan et al. 1991), suggesting the possibility of highly dynamic multisensory control of odor processing and perception.

While it has been demonstrated that multisensory associative cues can modulate POC odor processing and subsequent odor perception, little is known about the POC's selectivity and dynamics, that is, the temporal behavior during multisensory association. Here, we used an fMRI paradigm comprising pairings of a visual cue with an odor at different intensities. Specifically, this fMRI paradigm allowed us to determine, within subjects, whether visual cue‐evoked activity within the primary olfactory cortex and other odor memory‐related structures tracked the odor intensity of the preceding odor–visual cue association, or simply reflected odor–visual cue association alone. We demonstrated that a learned odor–visual pairing cue is related to the immediate visual‐evoked activity within the primary olfactory cortex, secondary olfactory structures, and hippocampus. Additionally, this effect was intensity dependent and generated no visual‐evoked odor perception. The results provide novel data and methods suggesting new avenues to explore odor–visual integration and show how multiple brain regions contribute to the construction of a unified odor percept (Wilson and Stevenson 2006).

## Material and Methods

### Participants

Nineteen healthy, nonsmoking, right‐handed young adults (mean age = 27.0 ± 6.0 years, 11 female) participated in the study. All participants were screened for any otorhinolaryngeal, neurological, psychiatric, central nervous system, and olfactory system disorders. Each subject's smell function was evaluated using the University of Pennsylvania Smell Identification Test (UPSIT) (Doty et al. 1984) and scored within the normal range (mean = 35.7, SD = 2.8). All participants provided informed consent to the study as required by the Pennsylvania State University College of Medicine Institutional Review Board.

### Olfactometer

The odorants were delivered with a fully automated MR‐compatible olfactometer (ETT LLC, Hershey, PA). Lavender was selected as the odorant of choice because it is known to have low trigeminal nerve stimulation and perceived universally as pleasant and familiar (Allen 1936; Patton 1960; Field et al. 2005). Odorants were delivered with moisturized compressed room air at a constant air flow rate of 8 L/min to both nostrils of the subject through a 3‐mm diameter Teflon tubing in the scanner. The lavender intensities were prepared by diluting lavender oil (Quest International Fragrances Co., Mount Olive, NJ) in 1,2‐propanediol (134368; Sigma, St. Louis, MO) to generate weak (0.032%), medium (0.10%), strong (0.32%), and very strong (1.0%) odor intensities. The odorant delivery was synchronized with MR image acquisition and visual breathing instructions.

### fMRI paradigms

**Figure 1:**
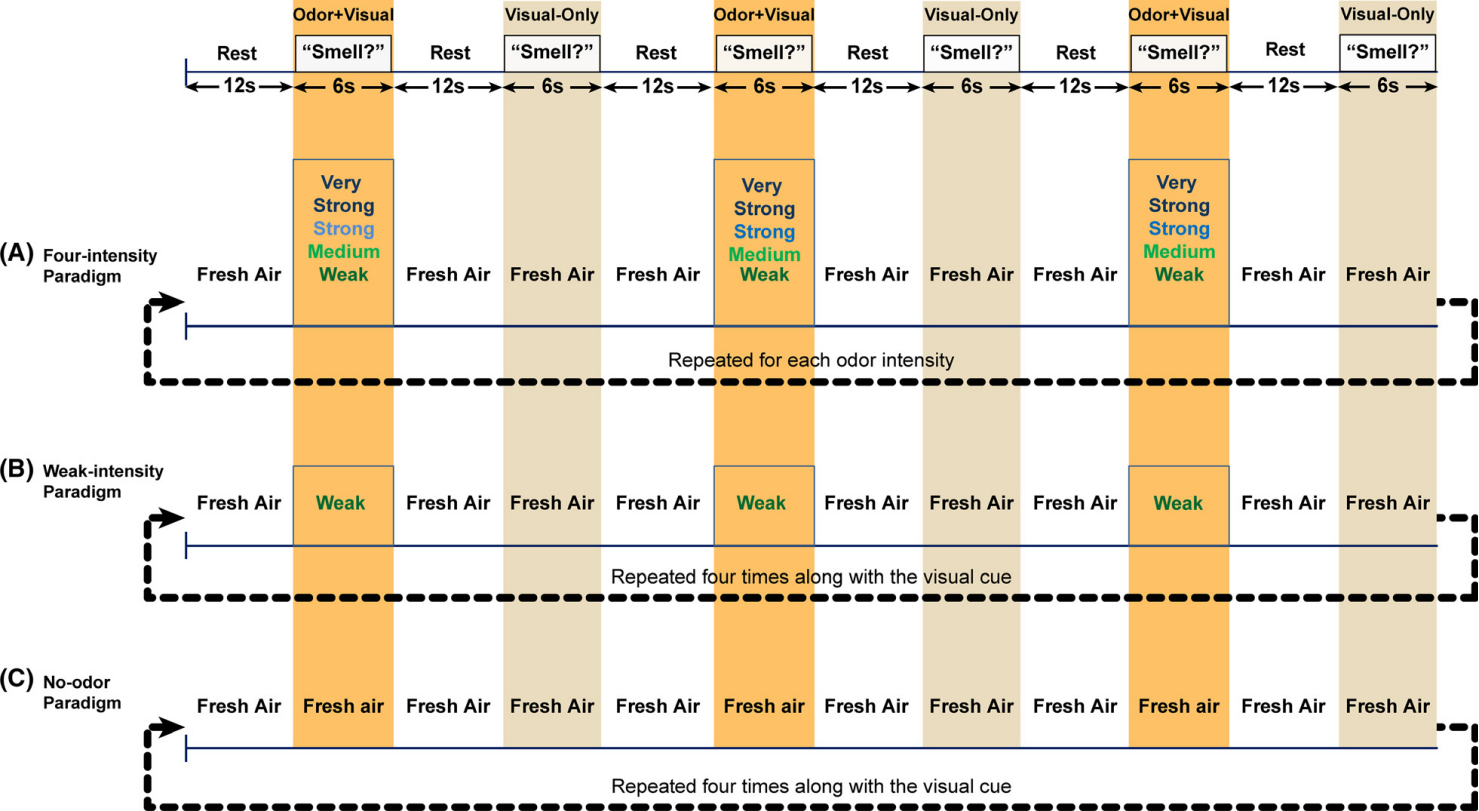
The fMRI imaging paradigms: (A) four‐intensity, (B) weak‐intensity, and (C) no‐odor paradigms. Each visual cue is a condition in all paradigms. The no‐odor paradigm followed the same pattern as the other two, except the visual cue “Smell?” which was not paired with an odor (i.e., fresh air). A constant airflow of 8 L/min was maintained throughout all conditions (including the “Rest”) so that no tactile or thermal cues were introduced. Participants had to perform a button press response to indicate the presence/absence of an odor during odor+visual and visual‐only conditions. The no‐odor paradigm served as the control experiment to investigate whether the visual‐only (i.e., visual cue) condition was influenced by odor–visual pairing.

To examine the odor learning behavior, three olfactory fMRI stimulation paradigms were administered to each subject in pseudorandomized order during a single imaging session. As shown in Figure 1, all three paradigms had the same visual stimulation timing structure paired with weak‐intensity odor, four‐intensity odor, and no‐odor olfactory stimulation as encoding steps, respectively. The visual stimulus was the word “Smell ?” written in white letters and presented against a black background during which participants had to perform a button press response for odor + visual (right index finger) and visual‐only (left index finger) conditions. The odorant concentrations for perceived odor intensities were determined using a systematic psychophysical experiment as described previously (Karunanayaka et al. 2014). In the four‐intensity paradigm, four different lavender odor intensities were presented sequentially in an incremental fashion (i.e., from the weakest to the strongest intensity) with each repeated three times. Odor presentation lasted for 6 sec and was followed by two conditions: 12 sec with a visual cue “Rest” and then 6 sec with the visual cue “Smell?”. A constant airflow of 8 L/min was maintained throughout all conditions in order to avoid tactile or thermal stimulation. Advantages of this experimental design, including its ability to overcome olfactory habituation have been discussed using a different dataset in detail elsewhere (Karunanayaka et al. 2014). All questions addressed in the current manuscript are nonoverlapping and discrepancies, if any, have been highlighted in the Discussion section.

The rationale to use the weak‐intensity paradigm in our experiments was due to a limitation of olfactory fMRI. Generally, subjects do not tolerate long and continuous olfactory fMRI scanning sessions. This made it impossible to increase the number of repetitions for each odor intensity (say six for better statistical power) within the four‐intensity paradigm. Therefore, in order to investigate whether visual cue‐evoked activity within odor‐related structures tracked the odor intensity we employed the weak‐intensity paradigm. Other innovative aspect of our design was the use of no‐odor paradigm as the control experiment. If the fMRI activity during the visual‐only condition was not influenced by odor–visual pairing, then we expect to observe that the percent signal change during the visual‐only condition is similar across all three paradigms.

The study coordinator carefully monitored the task compliance during fMRI scanning. Additionally, subjects were instructed not to sniff and their breathing patterns were monitored and recorded using a chest belt (Wang et al. 2013). Participants who had more than 95% response accuracy and normal breathing patterns were included in the current study. We also tested a strong‐intensity paradigm though found high intersubject variability in the BOLD signal (possibly due to significant habituation effects), and thus this paradigm was not considered for further analysis in this study.

The studies were performed on a Siemens Tim Trio 3T system. MR images of the entire brain were acquired using EPI with the following parameters: acceleration factor = 2, TR = 2000 ms, TE = 30 ms, flip angle = 90°, FOV = 220 mm × 220 mm × 120 mm, acquisition matrix = 80 × 80, number of slices = 30, slice thickness = 4 mm, and number of repetitions = 234. A T_1_‐weighted, high‐resolution anatomical image was also acquired from each subject for functional map overlay with the following parameters: TR = 2300 ms; TE = 2.98 ms; flip angle = 9°; FOV = 256 mm × 256 mm; matrix size = 256 × 256; slice thickness = 1 mm (no slice gap); number of slices = 160; voxel size 1 mm × 1 mm × 1 mm.

### Data processing and analysis

The fMRI images were realigned, coregistered, and normalized to the Montreal Neurological Institute brain template using SPM8 (Collins et al. 1998). The fMRI images were resliced into 2 mm × 2 mm × 2 mm voxels before applying a smoothing Gaussian kernel of 8 mm × 8 mm × 8 mm (full width at half maximum). Activation maps for the odor + visual and visual‐only conditions, and for each subject, were computed using event‐related generalized linear models (GLMs) and used in a second level random effect analysis to generate group activation maps. Since subjects had normal smell function, UPSIT scores were not included as covariates (variant of no interest) in the level 1 GLM analysis. Since there were no significant differences between odor intensities (in terms of hemodynamic response function [HRF]) during the four‐intensity paradigm, we investigated only the overall effect of odor + visual and visual‐only conditions by combining respective conditions in each odor intensity. The odor + visual and visual‐only conditions were compared using a paired *t*‐test. The intensity dependence of olfactory response was not investigated because there were only three repetitions for each odor intensity. We also investigated the hemodynamic response function for odor + visual and visual‐only conditions by convolving respective condition vectors of onset times with a finite impulse response function (IRF) with eight 2‐sec time bins (Goutte et al. 2000; Ollinger et al. 2001; Lindquist et al. 2009).

Spatial group ICA was used to investigate the brain networks that subserve odor‐related, associative learning behavior in our paradigms. This method can reveal chronoarchitectonically identified areas or functionally connected regions and generally provides additional information when compared to standard regression‐based fMRI data analyses techniques (Bartels and Zeki 2004; Karunanayaka et al. 2014). Additionally, under certain minimal assumptions the spatial and temporal behavior of each IC map can also be linked to a specific cognitive function (Duann et al. 2002; Calhoun et al. 2004; Karunanayaka et al. 2007).

In general, ICA methodology consists of two parts: (1) preprocessing steps such as realignment, normalization, smoothing, mean centering, and principal components analysis (PCA) both at individual as well as group levels, and (2) ICA decomposition and estimation using repeated runs of the FastICA algorithm followed by hierarchical agglomerative clustering (Hyvarinen 1999; Himberg et al. 2004). The IC validation, in terms of task relatedness, is determined by (1) the spectral power of each IC time course at the task frequency and (2) the phase of each IC time course relative to the task reference function (Schmithorst et al. 2006; Karunanayaka et al. 2010). Finally, a voxel‐wise random effects analysis (one‐sample *t*‐test) is performed on individual IC maps to determine the group IC maps.

Using IC time courses, hemodynamic response functions (HRF) and the respective single‐trial responses of each network were evaluated as described elsewhere (Eichele et al. 2008). This method entails estimating the HRF by forming the convolution matrix of the stimulus onsets and multiplying its pseudoinverse with the IC time course. This is followed by single‐trial estimation (i.e., beta) by fitting a design matrix containing predictors for the onset times of each trial convolved with the estimated HRF in the previous step, onto the IC time course.

Regions of interest (ROIs) analyses were performed to establish causal connections among the POC, insula, hippocampus, and the OFC during the four‐intensity paradigm. Respective ROIs were used to extract the mean percent signal change (averaged over the ROI) representing the blood‐oxygen‐level‐dependent (BOLD) response for a subsequent unified structural equation modeling (uSEM) analysis (Gates et al. 2011; Kim et al. 2007). FMRIB Software Library View (FSLview, Analysis Group, FMRIB, Oxford, UK) was used to perform the bilateral manual segmentation of the POC on a MNI template images (Vasavada et al. 2015). The POC included the anterior olfactory nucleus, olfactory tubercle, piriform cortex, anterior portion of the periamygdaloid cortex and amygdala, and anterior perforated substance (Wang et al. 2010). Other ROIs were obtained from the AAL‐segmented brain atlas (Tzourio‐Mazoyer et al. 2002). The previously published fMRI study of this paradigm only looked at the POC using a manually defined square ROI that encompassed the full extent of the POC located within the rostral–medial surface of the temporal lobe (Karunanayaka et al. 2014). Nevertheless, the uSEM method entails estimating causal influences (including lagged effects) among measured or latent variables (Bollen 1989; Bollen and Long 1993; Joreskog and Sorbom 1996; Tzourio‐Mazoyer et al. 2002; Gates et al. 2011).

In neuroimaging, uSEM is primarily used for effective connectivity estimation presumed to mediate directional influences of one neuronal system on another. The connectivity estimation is operationalized in terms of parameters in a neuronal model aimed at explaining observed brain activity dependencies (i.e., functional connectivity networks) (Friston 2011). Therefore, effective connectivity can be identified with the intuitive notion of coupling or directed causality that explicitly rests on a model of neuronal influence (Karunanayaka et al. 2007; Friston 2011). Additionally, uSEM was combined with the Group Iterative Multiple Model Estimation (GIMME) technique to identify the optimal olfactory network across participants that best describes the causal structure based on network element time behavior during the four‐intensity paradigm. GIMME iteratively identifies the causal connections that, if estimated, would offer the greatest statistical improvement for at least 75% of the individuals in the sample (Gates and Molenaar 2012).

## Results

### Rapid odor–visual association

**Figure 2:**
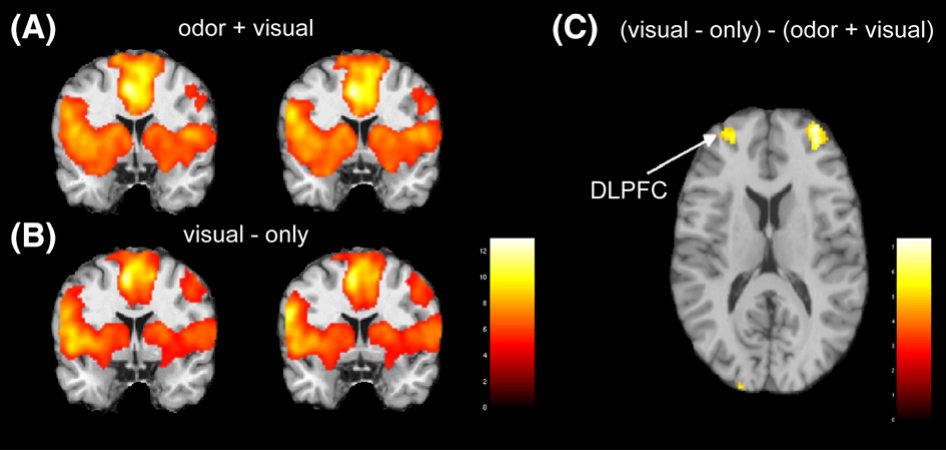
Group activation maps for the (A) odor + visual and (B) visual‐only conditions during the four‐intensity paradigm (*n* = 18, *P* < 0.0001, cluster size = 125). Both conditions show comparable activation patterns and suggest that the activation during the visual‐only condition was influenced by the odor + visual condition. (C) The statistical difference map between visual‐only and odor + visual condition (*n* = 18, *P* < 0.0001, cluster size = 100). Higher activation was detected in the dorsal lateral prefrontal cortex (DLPFCF) during the visual‐only conditions.

Subjects responded at better than 95% accuracy to the presence or absence of odor in the odor + visual and visual‐only conditions within each and across all three paradigms. Figure 2A and B show the brain activation patterns for the odor + visual and visual‐only stimulation conditions during the four‐intensity paradigm with corresponding activation clusters tabulated in Table 1. A striking observation is that the visual‐only stimuli elicit nearly identical activation patterns as the odor + visual stimuli in the primary olfactory cortex, insula, hippocampus, and orbitofrontal cortex. When this condition was compared to the odor + visual condition, no significant activation differences were detected in olfactory brain structures except in the dorsal lateral prefrontal cortex (DLPFC) (paired *t*‐test, *P* < 0.001 and Fig. 2C and Table 2). These results indicate that the activation in the olfactory system, produced by the visual‐only stimuli, was closely associated with the preceding odor + visual condition, suggesting a rapid associative learning process.

**Table 1:** Olfactory‐related brain structures and activation foci during the four‐intensity paradigm for the odor + visual and visual‐only conditions. The corresponding GLM map is shown in Figure 2A and B

Region	L/R	Volume		MNI Coordinates (*x*,* y*,* z*)		Peak *Z*		*P* values (FWE, uncorr)	
		Odor + visual	Visual‐only	Odor + visual	Visual‐only	Odor + visual	Visual‐only	Odor + visual	Visual‐only
Primary olfactory cortex	L	331	284	−28, 4, −20	−26, 2, −10	4.7	4.3	0.04, 0.001	–, 0.001
	R	263	226	24, 2, 18	20, 8, −16	4.6	4.3	0.06, 0.001	–, 0.001
Insula	L	741	751	−36, 16, 2	−48, −2, 0	5.1	5.9	0.006, 0.001	0.000, 0.001
	R	736	742	36, 22, 2	36, 22, 4	5.5	5.7	0.001, 0.001	0.000, 0.001
Orbitofrontal cortex	L	28	15	−30, 30, −8	−30, 30, −8	4.2	4.0	–, 0.001	–, 0.001
	R	–	5	–	20, 26, −12	–	3.4	–	–, 0.001
Hippocampus	L	10	12	−20, −4, −14	−22, −32, −4	3.7	3.7	0.6,0.0001	–, 0.001
	R	–	56	–	16, 28, −12	–	4.0	–	–, 0.001
Striatum	L	133	133	−16, 8, 0	−26, 6, 2	4.8	4.7	0.02, 0.001	0.032, 0.001
	R	69	68	20, 8, −4	16, 8, −2	4.6	4.0	0.05, 0.001	–, 0.001
Cingulate	L and R	4026	3607	−6, 6, 42	−8, 8, 44	6.1	5.9	0.001, 0.001	0.001, 0.001

L, left; R, right. MNI, Montreal Neurological Institute.

**Table 2:** Brain structures and activation foci for the difference between odor + visual and visual‐only conditions in the GLM maps shown in Figure 2B

Region	L/R	Volume	MNI Coordinates (*x*,* y*,* z*)	Peak *Z*	*P* values (uncorr)
Dorsal lateral prefrontal cortex	L	*155*	−28, 54, 14	4.6	0.001
	R	234	32, 60, 12	4.9	0.001
Primary visual	L	681	36, −60, 50	4.8	0.001
	R	144	54, 24, 28	4.5	0.001
Parietal cortex	L	–	*–*	–	–
	R	171	−36, −92, −2	4.5	0.001
Dorsal lateral prefrontal cortex	L	–	*–*	–	–
	R	408	54, 24, 28	4.5	0.0001

L, left; R, right. MNI, Montreal Neurological Institute.

If the olfactory system response to visual‐only cues after odor + visual pairing is due to associative learning, then the activation elicited by the visual‐only condition in the olfactory structures should depend on the characteristics of the odor presented during the preceding odor + visual conditions in the paradigm. To test this hypothesis, we performed an experiment with three different paradigms, each with different odor intensities (i.e., no‐odor, weak‐intensity, and four‐intensity paradigms) while keeping the visual stimulus the same. As mentioned earlier, the no‐odor paradigm effectively served as the control experiment. This is because if the presence of odors is not influencing subsequent visual‐only conditions, then the brain activity during the visual‐only condition should not be different across all three paradigms.

**Figure 3:**
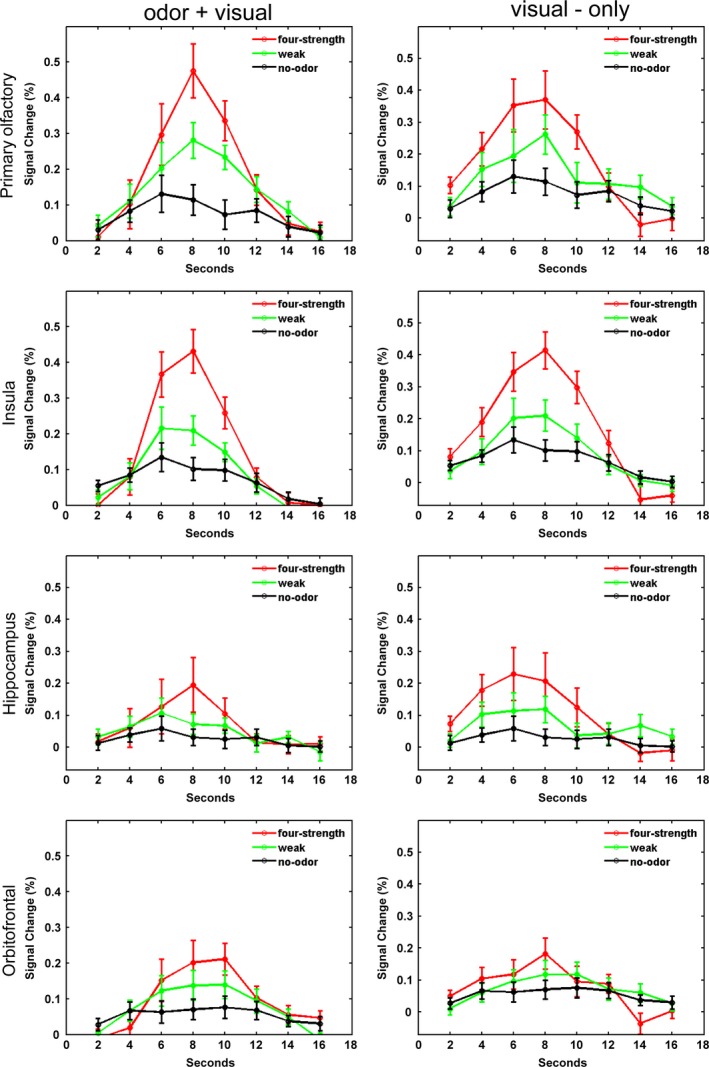
BOLD signal behavior in predefined ROIs during four‐intensity (red), weak‐intensity (green), and no‐odor (black) paradigms. The no‐odor paradigm had twice the number of visual‐only conditions compared to the four‐intensity and weak‐intensity paradigms. Half of them were treated as the odor + visual condition and the other half as the visual‐only condition, in the same order as those in the four‐ and weak‐intensity paradigms. All three paradigms produced a similar pattern of BOLD signal behavior during the odor + visual and visual‐only conditions in all ROIs. Comparisons were performed across the fourth time point data (i.e., 8 sec after stimulus onset) in estimated HRFs within and across paradigms.

Figure 3 shows the hemodynamic response function (HRF) of the BOLD signals (in terms of signal percent change) corresponding to odor + visual and visual‐only conditions in major olfactory structures (ROIs) during all three paradigms. First, the BOLD responses clearly exhibit a strong but nonlinear dependence with odor intensity during the odor + visual conditions. No significant activation could be detected during the no‐odor (i.e., control) paradigm where only fresh air was paired with the visual stimulus. The four‐intensity paradigm produced the strongest BOLD signal in all four brain structures during this condition (*P* < 0.01), while the weak‐intensity paradigm produced weaker BOLD responses. The BOLD signal difference between odor + visual and visual‐only condition were not significant during the weak‐ and four‐intensity paradigms (*P* > 0.05). When the visual‐only condition was compared between the four‐intensity and no‐odor paradigms, the four‐intensity paradigm produced significantly higher activity (*P* < 0.01). Except in OFC, the visual‐only condition was significantly higher in the weak‐intensity paradigm when compared to the no‐odor paradigms (*P* < 0.01).

These results suggest that activation in olfactory areas elicited by visual cues (i.e., visual‐only condition) may be a direct consequence of preceding odor + visual conditions in the respective paradigms. As such, the results indicate the possible associative learning mechanisms that are instantiated at the level of primary and secondary olfactory structures.

### Dynamics of odor–visual association

**Figure 4:**
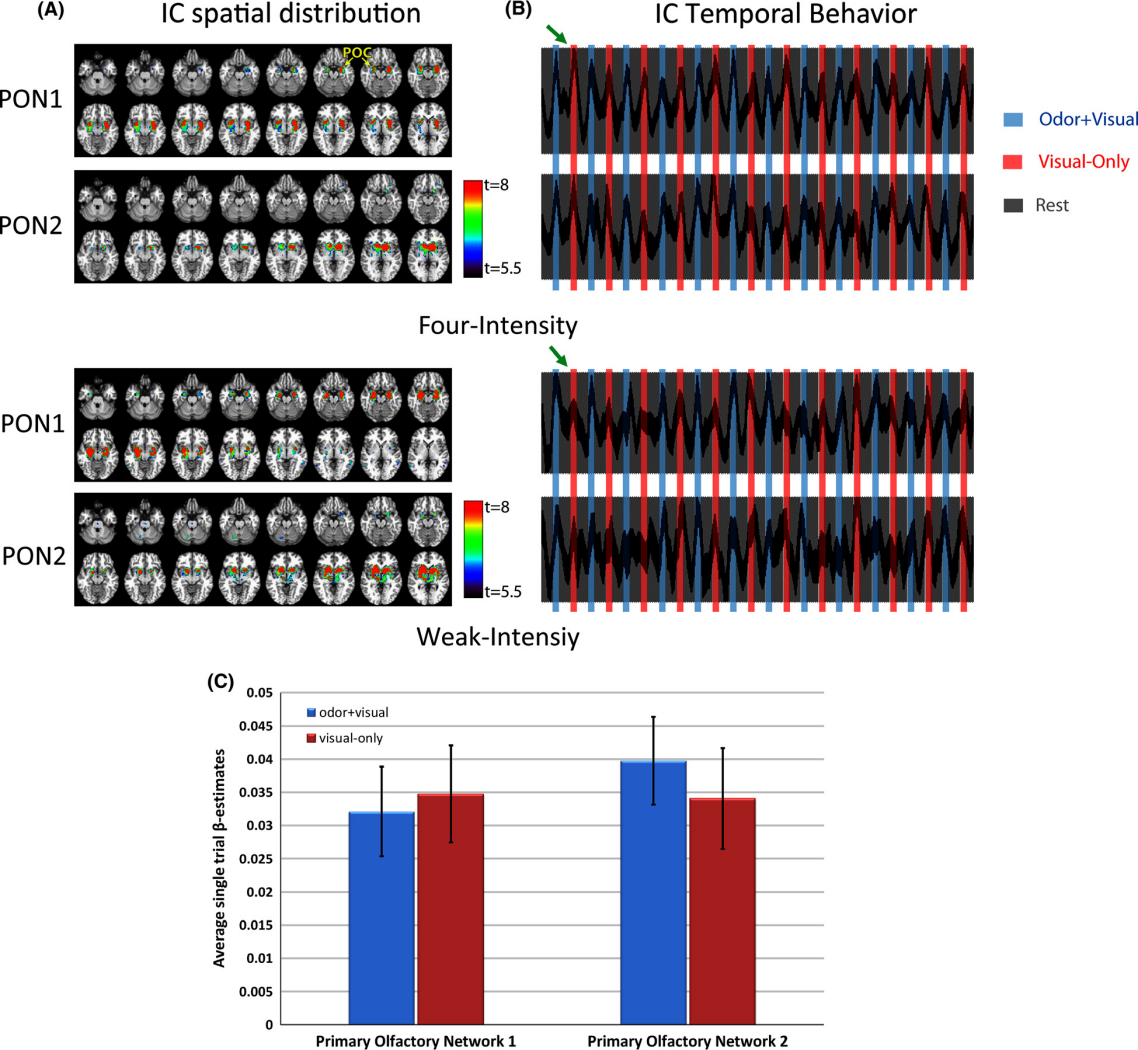
(A) Two task‐related IC maps (or components) encompassing bilateral primary olfactory cortex (POC), hippocampus, and striatum, etc., presumed to subserve odor‐related associative learning during the four‐ and weak‐intensity paradigms. All images are in neurological convention. Slice thickness = 2 mm. Threshold: *t* = 5.5–8 and corrected *P* < 0.05. PON: primary olfactory network. (B) Associated IC time courses for each IC map. Thickness of time courses is proportional to the intersubject variability. A rapid network response is indicated for the first visual‐only condition during the four‐intensity paradigm (green arrow). (C) Network responses for odor + visual and visual‐only conditions, in terms of average single‐trial *β* estimates during the four‐intensity paradigm. Networks showed no significant differences between the two conditions providing direct evidence for odor‐related associative learning even at the network level.

Since ICA can provide concurrent spatial as well as temporal information, we used this method to further investigate the dynamics of associative learning behavior that occurred during four‐ and weak‐intensity paradigms in our study. Figure 4 shows the IC maps and the corresponding time courses of two task‐related primary olfactory networks (primary olfactory network [PON] 1 and PON2) during the four‐ and weak‐intensity paradigms (Karunanayaka et al. 2014). Previously, we have identified five task‐related IC maps for this task, though our focus is on two that include primary and secondary olfactory structures (i.e., primary olfactory related; Karunanayaka et al. 2014). These IC maps show activation in bilateral POC, bilateral insula, bilateral hippocampus, orbital frontal cortex, and bilateral striatum (Table 3) agreeing with the results seen in the GLM analysis as shown in Figure 2. As clearly shown in the corresponding time courses, the visual‐only condition elicited a strong activation in both PON1 and PON2. Most interestingly, the activations were triggered by the very first visual‐only stimulation that was preceded by the odor + visual paring condition, demonstrating that the association of the visual cue with a specific odor stimulus in the olfactory system is immediately established by a single encoding (associative) event of a paired stimulus. No significant difference was found between the visual + odor and visual‐only conditions over the time course of the four‐intensity paradigm (paired *t*‐test, *P* > 0.05) in terms of average single‐trial *β* estimates in these two networks (Fig. 4C). Between the two paradigms, the correlation of BOLD signal during odor + visual and visual‐only conditions is the strongest in the four‐intensity paradigm. Also note that the degree of intersubject variability, as indicated by the dispersion of IC time courses, becomes higher in the weak‐intensity paradigm than that of the four‐intensity paradigm. This is likely due to the differences in the interplay of the habituation effect by the repetitive same odor‐intensity stimulation (Tabert et al. 2007).

**Table 3:** Brain structures and activation foci (maximum) for each IC component shown in Figure 4. The voxel size is 2 × 2 × 2 mm^3^

Network	L/R	Main brain regions	Peak *Z*		MNI Coordinates (*x*,* y*,* z*)	
			L	R	L	R
PON1	L and R	POC	12	12	−29, −1, −14	31, −1, −12
	L and R	Hippocampus	9.2	13	−25, −15, −20	29, −9, −14
	L and R	Putamen	10.3	17	−27, 7, −6	31, 7, −4
	L and R	Globus pallidus	9.0	12.4	−25, 3, −6	23, −3, −6
PON2	L and R	Putamen/Ventral striatum	9.2	10.9	−21, 11, 8	19, 7, 0
	L and R	Globus pallidus	8.9	8.4	−17, 7, 4	17, 1, 0
	L and R	Caudate	8.7	12	−11, 13, 10	19, −1, 20
	L and R	POC	6.4	9	−17, 7, −16	15, 13, −14
	R	OFC	–	7.2	–	31, 31, −18
	L and R	Thalamus	9.1	10.9	−7, −5, 8	3, −17, 16

POC, primary olfactory cortex; OFC, orbitofrontal cortex; PON, primary olfactory network; L, left; R, right. MNI, Montreal Neurological Institute.

### Causal structure of odor–visual association

**Figure 5:**
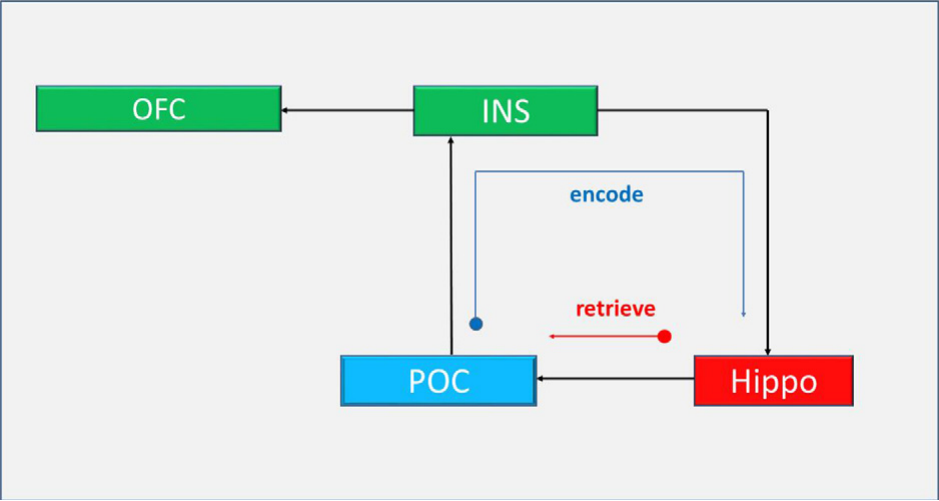
The optimal uSEM for odor learning during the four‐intensity paradigm. This model supports hippocampus as a binding structure that can maintain a distributed trace of a previously encountered odor (i.e., encoded) during the odor + visual condition. We propose that this structure can trigger activation in olfactory structures during the visual‐only condition as part of the retrieving process. As such, our model shows that odor processing involves not only POC but also OFC and INS. Only the contemporaneous connections are shown in this figure. POC, primary olfactory cortex; INS, insular cortex; OFC, orbitofrontal cortex; Hippo, hippocampus.

Unified SEM (uSEM) was used to investigate the effective connectivity of the olfactory system activation during the four‐intensity paradigm. Directed connections from POC to insula and then to OFC and hippocampus were identified in the optimal group model shown in Figure 5, which presumably may subserve associative learning processes as indicated by the blue arrow. As indicated in Table 4, a highly significant directed connectivity from hippocampus to POC in the model was also identified, which can be interpreted as a dominant effect during the “retrieval” process and indicated by the red arrow in Fig. 5. This is consistent with hippocampal involvement in this type of rapid associative learning behavior in primary olfactory cortex, insula, and orbitofrontal cortex (Gottfried 2010; Henke 2010). As such, the network connectivity under our paradigm reflects a combined trace of multisensory association (or integration) across olfactory structures which are retrieved during the visual‐only conditions.

**Table 4:** Average *β* estimates across all individuals for connections obtained for the uSEM in the group‐level search (Fig. 5). “% significant” indicates the percentage of individuals for whom the connection remained significant after accounting for individual‐level effects (obtained in the second stage of the GIMME search)

	Mean *β*	% Significant
Contemporaneous		
Hippo → POC	0.71	100%
INS → Hippo	0.4	83%
INS → OFC	0.38	100%
POC → INS	0.21	67%

## Discussion

This study provided evidence for a rapid learning response in the olfactory system wherein a conditioned visual cue (visual‐only condition) produced strikingly similar activation patterns in olfactory brain structures as the preceding odor + visual condition. The response in the olfactory cortical regions to the conditioned visual cue was modulated in accord with the odor‐intensity variations of the preceding odor + visual conditions. Specifically, pairing a visual cue with no odor (i.e., fresh air) during the preceding odor + visual conditions in the no‐odor control paradigm produced no significant visually evoked olfactory system activation. In contrast, the visual‐only condition following odor–visual association produced robust visual‐evoked olfactory system activations that were significantly different from the same condition in the no‐odor paradigm and tracked the intensity of the associated odor cue. Taken together, these data suggest a rapid, transient visual–odor association permitting visual cue activation of the olfactory pathway. The resulting visual‐evoked olfactory system response represents either a direct cross‐modal activation of the olfactory system or a learned olfactory expectation response evoked by the visual cues. The results further emphasize the robust multisensory control of olfactory cortical regions (Gottfried and Dolan 2003; Wesson and Wilson 2010; Maier et al. 2012).

In our previous ICA analysis of this fMRI paradigm, we reported significant differences between the odor + visual and visual‐only conditions in the second time‐bin values of the impulse response function (IRF) analysis (Karunanayaka et al. 2014). This discrepancy can be attributed to two reasons: the use of a (1) completely different data set and (2) manually drawn coarse ROI (square) to extract BOLD signal from the POC. However, as reported here, the previous study also generated slightly lower BOLD activity during the visual‐only condition in the POC when compared to the visual + odor condition (Karunanayaka et al. 2014).

Despite the visual‐evoked olfactory system activation that is nearly identical to the preceding visual + odor condition, no subjects reported the perception of an odor in any of the visual‐only conditions regardless of prior odor association. During the visual‐only condition a significantly higher activity is seen in the DLPFC compared to the visual + odor condition (Fig. 2C and Table 2). DLPFC is known to be involved in executive functions, such as working memory, inhibition, and abstract reasoning. The current paradigm seemed to evoke all these functions as the subjects saw the visual cue while the anticipated odor was not detected. Under such condition, higher cognitive functions may have been required during the no‐odor condition beyond the primary olfactory processing. Therefore, the higher activation in prefrontal regions during the visual‐only condition may subserve executive control related to working memory and attention as part of the recall process of associative learning in our paradigms.

The associative visual activation extended beyond POC to include the OFC, hippocampus, and insula. Each of these regions receives strong direct or indirect olfactory inputs and have been shown to be involved in multisensory perception such as flavor (Royet et al. 2001; Cerf‐Ducastel and Murphy 2006; Veldhuizen et al. 2010; Small 2012; Wu et al. 2012). Visual cues play an important role in both odor and flavor perception, presumably due to learned past multisensory associations (Zellner et al. 1991). Visual cues have also been demonstrated to modify human olfactory system activity, with congruent odor–visual cue pairings inducing the greatest activation (Gottfried and Dolan 2003). Areas most strongly affected by congruent odor–visual association in the Gottfried and Dolan's study, and most predictive of perceptual ratings, included the OFC and hippocampus—regions also responsive to associative effects in the present study.

Most, if not all, previous work on visual association of odor‐evoked activity and odor perception have relied on stable, naturally occurring odor–visual cue pairings, learned through a lifetime of interaction. Here, we provide evidence for a new type of association or learned expectation that appears to be rapid and highly dynamic. It is rapid because the very first visual‐only trial (Fig. 4) elicited activation in olfactory structures that was very similar to the activation elicited by subsequent visual‐only condition. In this aspect, these olfactory structures seem to behave similar to a working memory system. Specifically, the magnitude of the visually evoked olfactory system activation was intensity specific (i.e., it tracked the intensities under which odor–visual association was established in the paradigms). Varying suprathreshold odor intensity caused significant changes in BOLD signal across paradigms in the POC, insula, hippocampus, and OFC. Because our results demonstrate remarkable precision and efficiency in the acquired associative information, we argue that this association (or expectation) is much more complicated than a simple heteromodal representation of a visual stimulus.

Rapid olfactory system plasticity has been demonstrated in rodent models at the single‐unit level (Wilson 2003), though multisensory plasticity at this level has not been examined to our knowledge in the rodent olfactory system. One caveat of this study is the fact that it is impossible to determine for sure whether the activation during visual‐only trials is truly caused by the associative visual stimulus only (i.e., cross‐modal activation) or rather “expectation/priming” in the olfactory system due to the structure of trials in our paradigms. However, the observed increased DLPFC activation during the subsequent visual‐only cues tends to support the former interpretation as its function in memory and learning is well established. Further work will be required to describe both the time course of this associative behavior and whether the nature and quality of the visual and olfactory cues could influence this effect.

The two highly task‐related brain networks (PON1 and PON2) identified by ICA during odor stimulation paradigms support anatomical separation and functional specialization of olfactory‐related brain regions (Karunanayaka et al. 2014). The two IC maps shows clear distinction in functional specialization for anterior and posterior aspects of the primary olfactory‐related brain structures (Gottfried 2010). The PON1 encompasses hippocampal activation that may be related to implicit and explicit memory demands under the stimulation sequence in our paradigms (Schendan et al. 2003; Henke 2010). The IC time courses shown in Figure 4 clearly exhibit olfactory task‐related behavior during visual‐only conditions supporting associative learning in olfaction. The weak‐intensity paradigm time course showed relatively high intersubject variability potentially indicating odor sensitivity effects within the study cohort. The estimated HRFs for odor + visual and visual‐only conditions during the four‐intensity paradigm are similar in shape and the network responses, in terms of average single‐trial *β* estimates, are not significantly different. Thus, our results clearly support odor‐related learning during the four‐intensity paradigm at the brain network level. Since learning is part of olfactory processing, our results show that olfaction does not consist of a one‐sided transform from sensory content to neural representation. As such, the multifaceted neural representation of odors combine to show that olfactory perception is indeed an active process. Thus, the POC, in sync with other olfactory structures, may actively mediate the process of olfaction not only by coding odorant quality, but also by coding prior information about stimuli, thereby determining when the attentional focus shifts from merely breathing in air to smelling the world around us (Wilson 2003; Zelano et al. 2005).

Accordingly, the model established by the uSEM in Figure 5 exhibits probable causal connections among the POC, insular, hippocampus, and the OFC during the four‐intensity paradigm. The final model indicates that the activity in POC is directed to insular cortex and then to OFC and hippocampus (Savic et al. 2000). Although there are strong direct and indirect anatomical connections between POC, OFC, and insula, the existence of such connections between cortical regions is not a necessary condition to be functionally connected (Mesulam and Mutshon 1985). As such, a directed functional connection from insula to hippocampus and from hippocampus to the POC was identified in the group model. Since the model derived by uSEM reflected the average effect on the dynamics of the BOLD signals in both the encoding and the retrieval processes, we propose to decompose this model into two hypothetical temporal sequences: one to be taken as the driving effect for the visual‐only (retrieval) condition. This derives support from a taste aversive paradigm where insular cortex was implicated in integrating environmental and interoceptive information to help form efficient and rapid memory traces (Guzman‐Ramos and Bermudez‐Rattoni 2012). Thus, our data for this paradigm support an associative learning model with the hippocampus initiating a rapid activation of olfactory structures previously engaged in odor–visual encoding. Along these lines, Gottfried et al. (2004) have proposed the hippocampus as a binding structure that can maintain a distributed trace of previously encountered odors across sensory‐specific regions. Our results support and extend such a model even further that the visual cues preceded by paired odor stimuli may trigger hippocampal activation that subsequently stimulates the olfactory network. This process is highlighted in our ICA time courses where PON1 (encompassing the hippocampus) activity is clearly present during visual‐only conditions (Fig. 4). The notion that the hippocampus reconstructs the entire trace (odor‐object) across sensory regions is also supported by the activation in primary olfactory cortex during the visual‐only condition (Figs. 3 and 4).

### Limitations of the study

This study employed the no‐odor paradigm as the control experiment. Findings can be further strengthened by employing an experimental design that uses within paradigm control conditions to investigate differential fMRI activity between the odor + visual and visual‐only conditions. Such a design can make direct comparisons to differentiate common (e.g., visual stimulation, motor responses, perceptual, cognitive, and motivational) as well as different aspects that are unique to each condition. Furthermore, the number of trials (three) for each intensity within the four‐intensity paradigm was not sufficient to test whether the fMRI signal varies with odor intensity for both the odor + visual and visual‐only conditions. Event‐related fMRI designs with relatively long interstimulus intervals (ISI) may strengthen these findings by facilitating better differentiation between associative leaning processes and after effects of olfactory stimulation. We also note that care should be taken when assigning directional influence (or causality) based on the temporal ordering of BOLD signals among ROIs. This is because delays in BOLD response to stimuli can vary across regions and subjects (Goutte et al. 2000; Ollinger et al. 2001). Therefore, the intuitive notion of coupling or directional influence during the four‐intensity paradigm exclusively rests on the proposed neuronal model for associative learning in this article. Finally, future studies with large number of subjects as well as different odorants are needed to confirm our findings and to better understand multisensory association in the olfactory cortex.

In summary, the results of this study demonstrated activation of regions involved in olfactory processing and memory by a visual stimulus previously paired with an odor. The efficacy of the visual stimulus to evoke olfactory system activity was high. This activation intensity followed the activity (or intensity) pattern evoked by the most recent odor–visual paring. Subjects did not report perceiving an odor during visual‐only conditions in all three paradigms. The same visual stimulus not paired with an odor did not evoke olfactory pathway activity. Our results add to the growing literature on modulatory effect of olfactory system activation by nonolfactory cues. As such, olfactory structures should be considered as active participants in a complex odor and memory processing network. In addition, the results emphasize the multisensory nature of odor perception, and add an important dynamic component to this multisensory convergence. Since odor–visual integration is highly influenced by congruent (or incongruent) combinations of odors and words (or pictures), future studies should investigate how such combinations modify learning in the olfactory system (Gottfried and Dolan 2003; Olofsson et al. 2014). Deciphering the underlying mechanisms of odor learning will further our understanding of olfactory information processing and will lay the foundation to delineate the specific contributions of olfactory cortex to odor perception (Anderson and Sobel 2003; Gottfried 2010).

## Conflict of Interest

The authors do not report any conflict of interest.
